# Editorial for the Special Issue on Nonlinear Photonics Devices

**DOI:** 10.3390/mi11080760

**Published:** 2020-08-07

**Authors:** Luigi Sirleto, Giancarlo C. Righini

**Affiliations:** 1 National Research Council (CNR), Institute of Applied Sciences and Intelligent Systems (ISASI), Via Pietro Castellino 111, 80131 Napoli, Italy; 2 National Research Council (CNR), Institute of Applied Physics (IFAC) “Nello Carrara”, Via Madonna del Piano 10, 50019 Sesto Fiorentino, Florence, Italy

There is some incertitude on the creation of the term “photonics” and some ambiguity about its frontiers (and differences with respect to optoelectronics and electro-optics). Many authors consider the French scientist Pierre Agrain as the “father” of photonics, as of 1967, even if it would be more correct to refer to an almost simultaneous invention of the word by a group of French physicitsts working in lasers and fiber optics and by a Dutch group of high speed photography specialists. The first appearance of this word was apparently in 1952 [1]. A very interesting analysis of the use of the term photonics, embracing history, philosophy, and sociology of science, was published recently [2].

What is sure is that “photonics” was increasingly used after the broadening of laser applications, and nowadays there is a rather general consensus on the definition given in the web page of UNESCO 2015 International Year of Light and Light-Based Technologies: “Photonics is the science and technology of generating, controlling, and detecting photons, which are particles of light” [3].

In most cases, the response of a material to an optical field is linear (i.e., the strength of the response is proportional to the strength of the optical field), but all the way back in the second half of the XIX century, John Kerr, in Glasgow, observed effects that were proportional to the square of the applied field. The field of nonlinear optics, however, started to grow up only after the invention of the laser, when intense light sources became easily available. The seminal studies by Peter Franken [4] and Nicolaas Bloembergen [5], in the 1960s, paved the way to the development of today’s nonlinear photonics, the field of research which encompasses all the studies, designs, and implementations of nonlinear optical devices which can be used for the generation, communication, and processing of information.

Ten years after Franken’s paper, Anderson and Boyd performed the first nonlinear optics experiment in waveguides: as for bulk nonlinear optics, it dealt with frequency conversion, namely, second harmonic generation (SHG) in gallium arsenide (GaAs) waveguides [6]. It became soon clear that waveguides would offer fundamental advantages for nonlinear optics due to the intrinsic radiation confinement, leading to high optical power densities over long propagation distances.

Of course, the general trend of science towards the nano-world has also influenced the development of photonics, which started from waveguides at micrometer scale, going through microphotonics structures, and finally coming to nanophotonics. In the last few decades, with the development of integrated and nano-optics, biophotonics, quantum, and free-space optical communication, the concept of “photonics” acquired a broader sense. Nowadays, “photonics” is used almost synonymously with the term “optics,” referring equally to both science and applications, while nonlinear optical phenomena, and devices based on them, play a key role both in the knowledge of the matter and in many applications of photonics. This justifies the continuation of fundamental studies, and the search for new or advanced materials—with higher nonlinear coefficients and/or better overall properties.

The goal of identifying an efficient device integration platform is another hot issue: it would enable the development of low-cost and reliable devices and systems, wherein nonlinear phenomena may find new or more effective applications in areas such as all-optical switching, all-optical signal processing, and quantum photonics. The use of nonlinear effects in optical waveguides and microcavities is also at the forefront of this research.

This field attracts huge attention, as confirmed by a search made by using the Clarivate Web of Science: almost 200,000 papers were published which refer to the topic “nonlinear optic*”. Over 36,000 papers with the same keyword were published in the last four years (2015–2018), and over 17,000 used the keyword “nonlinear photonic*”.

The present Special Issue (SI) of *Micromachines* journal, titled “Nonlinear Photonics Devices,” aims at highlighting the current state of the art, some recent advances, and some perspectives for further development. Fundamental and applicative aspects have been considered, with special attention to the hot topics that could lead to technological and scientific breakthroughs. Contributions were solicited from both leading researchers and emerging investigators. As a result, this SI contains six reviews and six research articles.

The first group of articles has to do with nonlinear optical phenomena in optical waveguides, of fiber and integrated optical types. Going to the nanoscale level, the paper by Roland et al. [7] investigates the nonlinear properties of nanowire and nanorib waveguides in AlGaAs, which have the advantage of exhibiting an adjustable modal birefringence and supporting phase-matched frequency mixing in the whole AlGaAs transparency range, even close to the gap. In particular, the experimental performances and drawbacks of two different designs (a nanowire in straight or snake-shaped configurations, and a nanorib waveguide) of AlGaAs suspended nonlinear waveguides are compared. The authors conclude that, while the optical performances are almost identical for the two designs, the nanorib exhibits far better mechanical properties.

Optical fibers are often exploited for non-linear photonic devices due to their higher order intrinsic non-linear susceptibility χ(3): third harmonic generation (THG), self-focusing, and four-wave mixing (FWM) are some examples of the studied effects. SHG, on the contrary, would not be allowed in silica fibers, due to the absence of intrinsic second-order properties in centrosymmetric materials. This limitation has been overcome by the introduction, almost 30 years ago, of the technique of thermal poling. The article by De Lucia and Sazio [8] focuses on the logical and chronological development of 2D numerical models, with the aim of explaining in the best possible dynamics of evolution of the poling process. The authors have also identified the single-anode configuration as the most effective method for thermal poling, in terms of both the absolute value of the created quadratic non-linearity and of simplification of the fabrication constraints.

Another important nonlinear effect in optical fibers is due to inelastic-scattering, in which the optical field transfers part of its energy to the nonlinear medium, thereby inducing stimulated effects such as stimulated Brillouin scattering (SBS) and stimulated Raman scattering (SRS). Either of those types of stimulated scattering process can be used as a source of gain in the fiber. The article by Sirleto and Ferrara [9] reviews the state of the art, achievements, challenges, and perspectives of fiber Raman amplifiers (FRAs) and lasers (FRLs). FRAs are now widely used in fiber optic communications, in order to respond to the growing demand in terms of transmission capacity: the dramatic increase in bandwidth requirement has ruled out the use of erbium-doped fiber amplifiers (EDFAs), leaving fiber Raman amplifiers as the key devices for future ultra-high-capacity systems. FRLs, on the other hand, provide a very attractive option in the field of high-power fiber lasers. Nowadays, commercially available fiber-based Raman lasers can deliver output powers in the range of a few tens of Watts in continuous-wave operation, with high efficiency and broad gain bandwidth, covering almost the entire near-infrared region. The development of integrated RLs is reviewed in another paper, wherein Ferrara and Sirleto [10] describe the transition from the all-silicon Raman laser realized in 2005, based on a single-mode rib waveguide containing a reverse-biased p-i-n diode structure and fabricated on a standard silicon-on-insulator (SOI) substrate, to the current interest toward Si microphotonic structures based on photon confinement effects (nanocrystal waveguides, nanowires, and nanocavities).

Resonating structures, especially at microscale and nanoscale, are very attractive, due to the small volume and consequent high power density of the optical field, which gives higher strength to the nonlinear phenomena. Nonlinear photonics in resonators are the subject of another group of papers in the present SI. Ricciardi et al. [11] discussed the advances that occurred since it was shown that quadratic χ(2) processes can lead to direct generation of optical frequency combs in cw-pumped quadratic nonlinear resonators. Recently, direct generation of quadratic frequency combs has been demonstrated also in chip-scale, lithium niobite, periodically-poled, linear waveguide resonators and in whispering-gallery-mode (WGM) resonators. In this study, the authors analyzed and experimentally demonstrated comb generation in two configurations: a SHG cavity, where combs were generated both around the pump frequency and its second harmonic, and a degenerate optical parametric oscillator, where combs were generated around the pump frequency and its subharmonic. It may be worth noting that optical frequency combs are now attracting interest as sources of complex quantum states of light for high-dimensional quantum computation.

Nonlinear effects in solid and hollow microspherical WGM resonators (WGMRs) are reviewed in the paper by Frigenti et al. [12]. These structures are easy to fabricate and exhibit a very high quality factor Q; they are excellent platforms to understand how light, sound, and matter interact. Nonlinear photonic effects can be easily generated, and their very dense mode spectra allow one to efficiently fulfill the phase-matching conditions required for parametric and hyper-parametric interactions. This review describes Kerr effects in silica and hybrid (silica sphere with organic coating) WGMRs, including third-harmonic generation, third-order sum-frequency generation, frequency combs, Kerr switching, and two-photon fluorescence. Stimulated Raman scattering and stimulated Brillouin scattering, and combinations of other nonlinear phenomena, such as four-wave-mixing, are also discussed.

With the emergence of accurate nanofabrication techniques, there is interest in exploring nonlinear optical effects at a scale comparable to, or much less than, the incident light wavelength. At the nanoscale, interesting regimes for nonlinear optics emerge, in which the resonant optical interaction, due to frequency-selective light scattering or light coupling into and out of the structures, becomes significant. The resonant effects lead to a build-up of electric field inside or in the vicinity of the structure, resulting in enhancement of the nonlinear optical effects. The review article by Raghunathan et al. [13] provides an overview of this emerging field in dielectric-based sub-wavelength periodic structures to realize efficient harmonic generations, wavelength mixers, optical switches, etc. The structures considered here are broadly classified into guided-mode resonant structures and resonant metasurfaces; reference is made, for instance, to 1D gratings, 2D arrays of nanodisks, bar-nanodisk structures, asymmetric bar dimers, asymmetric rectangular unit-cells, and disordered nanodisk arrays. The basic physical mechanisms, the various nonlinear phenomena, and their applications are discussed too.

Exploiting at the best the photonic nonlinear effects requires a careful choice of structures and materials. Thus, some papers in this SI present a detailed analysis of these aspects. Bruno et al. [14] have studied thin films of epsilon-near-zero (ENZ) materials, such as transparent conductive oxides, including aluminum-doped zinc oxide (AZO) and indium tin oxide (ITO). In their paper, they demonstrate, both theoretically and experimentally, that a broadband coherent perfect absorption (CPA) based on light-with-light modulation may be achieved in these films. By using Kerr optical nonlinearities, the visibility and the peak wavelength of the total energy modulation can be dynamically tuned. The coherent control of the absorption in ENZ media may open a route towards technologies such as optical data processing or devices that require efficient light absorption and dynamical tunability.

The investigation of linear and nonlinear intersubband optical properties of quantum dots (QDs), which are of a great interest for integrated quantum photonic technologies, is the subject of the paper by Baira et al. [15]. Recently, GeSn has been shown to have comparable properties to III–V materials, while being compatible with complementary metal-oxide semiconductor (CMOS) technology. In this paper, the effects of an applied electric field on the electron-related linear and third-order nonlinear optical properties are evaluated numerically, with the aim of helping future realizations of CMOS-compatible, nonlinear optical devices. Pyramidal GeSn quantum dots with different sizes are considered. The results show that the transition energies and the transition dipole moment, particularly for larger dot sizes, are altered by the electric-field-induced electron confining potential profile’s modification.

Gallium arsenide has been widely used in photonic applications. Recently, it has also been proven that, due to its very high refractive index, nanostructures, such as GaAs nanowires, are able to effectively guide light by using leaky waves; this may lead to different applications as emitters or even as laser sources. Belardini et al. [16] have shown that glancing angle deposition of gold on GaAs nanowires induces a symmetry breaking that leads to an optical circular dichroism (CD) response that mimics chiral behavior. The presence of extrinsic chirality can have applications in different fields, including the ability to generate photons in a second-harmonic field, while selective pumping with circular polarized light could boost the processes of circular polarized photon generation or absorption. Geometric resonance that can be finely tuned by changing the diameter of the nanowires, is an essential feature in this extrinsic chiral behavior.

Periodically-poled lithium niobate (PPLN) is a material widely exploited for the implementation of nonlinear optical devices, both in bulk and in integrated optical format. Su et al. [17] used PPLN and MgO-doped PPLN to generate pure state photon triplets by cascaded second-order spontaneous parametric down-conversion (SPDC). Through numerical simulation, the most suitable parameters, in terms of pump duration and crystal length, were identified to eliminate the frequency correlation between the photon pairs in each SPDC process. Quantum interference is vital for quantum information science, since it is not only the basis of quantum manipulation technology, but is also an important tool for implementing quantum computing and quantum communication. The preparation of three photons with hyperspectral purity in the telecommunication C band is critical for research into quantum information processes and for applications.

Finally, another application of nonlinear phenomena, concerning the development of imaging techniques that are capable of reconstructing the full-wave properties (amplitude and phase) of arbitrary electromagnetic field distributions, is discussed in the paper by Gongora et al. [18]. Interestingly, the direct detection of the field evolution is achievable at terahertz (THz) frequencies thanks to the availability of the time-domain spectroscopy (TDS) technique. Such a capability, coupled with the existence of specific and distinctive spectral fingerprints in the terahertz frequency range, are critical enabling tools for advanced applications; a promising alternative to TDS imaging arrays is single-pixel imaging, or ghost imaging (GI). In this paper, the key advantages and practical challenges in the implementation of time-resolved nonlinear ghost imaging (TIMING) are discussed. TIMING combines nonlinear THz generation with time-resolved time-domain spectroscopy detection. The reported results establish a comprehensive theoretical and experimental framework for the development of a new generation of terahertz hyperspectral imaging devices.

Overall, this collection of scientific articles presents and discusses some interesting research topics in nonlinear photonics. It is our wish that this Special Issue will serve as a stimulus for students and researchers to further expand the potential of nonlinear photonics devices, via fundamental investigations and practical applications.

We would like to thank all the authors for their submissions to this special issue; we really have appreciated their contributions. We also thank all the reviewers for dedicating their time and helping to ensure the quality of the submitted papers. Last but not least, we are grateful to the staff at the editorial office of *Micromachines*—in particular to Mr. Dikies Zhang—for their efficient assistance.
